# Generation of Mature *Toxoplasma gondii* Bradyzoites in Human Immortalized Myogenic KD3 Cells

**DOI:** 10.21769/BioProtoc.4916

**Published:** 2024-01-05

**Authors:** Deborah Maus, Blake Curtis, David Warschkau, Estefanía Delgado Betancourt, Frank Seeber, Martin Blume

**Affiliations:** 1 Metabolism of Microbial Pathogens (P6), Robert Koch Institute, Berlin, Germany; 2 Mycotic and Parasitic Agents and Mycobacteria (FG16), Robert Koch Institute, Berlin, Germany; 3 Department of Microbiology and Molecular Medicine, Faculty of Medicine, University of Geneva, Geneva, Switzerland; 4 Research School of Chemistry, The Australian National University, Canberra, Australia

**Keywords:** *Toxoplasma gondii*, Tissue cysts, Bradyzoites, Cell culture, Human myotubes

## Abstract

*_Toxoplasma gondii_* is a zoonotic protozoan parasite and one of the most successful foodborne pathogens. Upon infection and dissemination, the parasites convert into the persisting, chronic form called bradyzoites, which reside within cysts in muscle and brain tissue. Despite their importance, bradyzoites remain difficult to investigate directly, owing to limited in vitro models. In addition, the need for new drugs targeting the chronic stage, which is underlined by the lack of eradicating treatment options, remains difficult to address since in vitro access to drug-tolerant bradyzoites remains limited. We recently published the use of a human myotube-based bradyzoite cell culture system and demonstrated its applicability to investigate the biology of T. gondii bradyzoites. Encysted parasites can be functionally matured during long-term cultivation in these immortalized cells and possess many in vivo–like features, including pepsin resistance, oral infectivity, and antifolate resistance. In addition, the system is scalable, enabling experimental approaches that rely on large numbers, such as metabolomics. In short, we detail the cultivation of terminally differentiated human myotubes and their subsequent infection with tachyzoites, which then mature to encysted bradyzoites within four weeks at ambient CO2 levels. We also discuss critical aspects of the procedure and suggest improvements.

Key features

• This protocol describes a scalable human myotube-based in vitro system capable of generating encysted bradyzoites featuring in vivo hallmarks.

• Bradyzoite differentiation is facilitated through CO_2_ depletion but without additional artificial stress factors like alkaline pH.

• Functional maturation occurs over four weeks.

**Graphical overview**


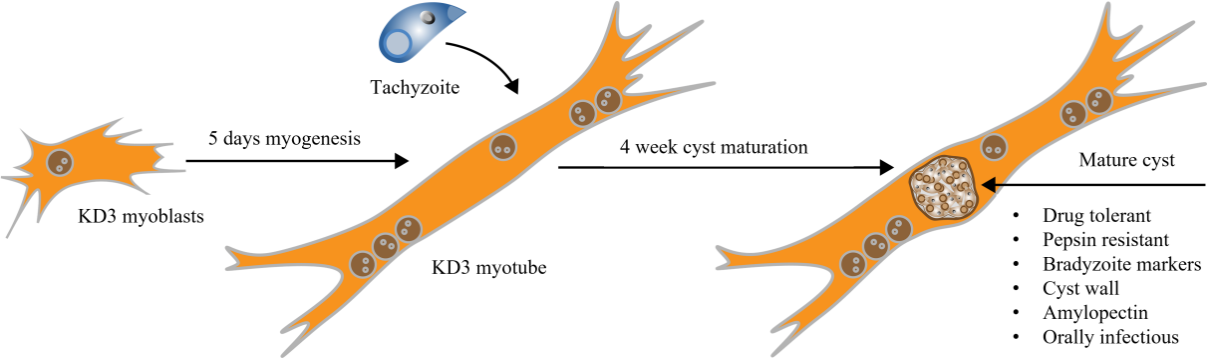


## Background

The obligate intracellular parasite *Toxoplasma gondii* infects nearly all warm-blooded animals and an estimated quarter of the human global population (Molan et al., 2019). The World Health Organization ranked *T. gondii* to be an important and common food-borne protozoan, with one of the highest disease burdens worldwide (Torgerson et al., 2015). It commonly manifests as an opportunistic pathogen in AIDS patients and other immunocompromised individuals (Luft and Remington, 1992). In contrast, most healthy individuals can effectively control the acute infection through their immune system (Denkers and Gazzinelli, 1998). Semi-dormant bradyzoites can persist for the duration of the host’s life in cysts within mainly the brain or muscle tissue, being resistant to current treatment options (Dubey, et al., 1998; Watts et al., 2015; Barrett et al., 2019). Bradyzoites can reactivate if the immune pressure abates (e.g., via AIDS, transplantation, etc.) leading to the emergence of severe clinical manifestations. Tissue cysts are a major source of transmission via the consumption of undercooked meat from infected animals (Gabriël et al., 2022). Hence, the investigation of bradyzoite biology including chemotherapy development is much needed.

In the past, corresponding research efforts were hindered by the lack of an appropriate in vitro model. Either isolated cysts from mice brain homogenates or functionally immature bradyzoites derived from fibroblast cell cultures were used. Many traits of bradyzoites, such as drug-tolerance mechanisms, remain challenging to study in vitro. Spontaneous conversion of tachyzoites to bradyzoites has been characterized in murine C2C12 myotubes (Guimarães et al., 2008; Ferreira-da-Silva et al., 2009; Swierzy and Lüder, 2015). We recently showed that immortalized human myoblasts (called KD3) are also suitable for long-term bradyzoite culture (Christiansen et al., 2022). KD3 myoblasts harbor transgenes for mutated cyclin-dependent kinase 4, cyclin D1, and a human telomerase reverse transcriptase (hTERT), resulting in the immortalization of these primary subcutaneous muscle cells (Shiomi et al., 2011). In differentiated KD3 myotubes, cystogenic strains of *T. gondii* can be matured over a period of four weeks, during which the cysts develop structural and functional traits of in vivo cysts (Christiansen et al., 2022) and express bradyzoite and cyst markers, such as BAG1, CC2, glycans, a discernible cyst wall, as well as amylopectin granules. In addition, the cysts become tolerant to the antifolates pyrimethamine and sulfadiazine and are orally infectious to mice. They gradually develop resistance to pepsin digestion and survive storage at 4 °C and exposure to 55 °C (Christiansen et al., 2022).

With this protocol, we aim to facilitate the use of in vitro *T. gondii* cysts in studies to reduce animal experiments and to increase the exploration of this comparatively understudied form of *T. gondii*.

## Materials and reagents

**Cell lines**

1. BJ-5ta human foreskin fibroblast (HFF) cells (ATCC CRL-4001)
2. *Toxoplasma gondii* Prugniaud- tdTomato (Chtanova et al., 2008) or any other cystogenic strain
3. KD3 myoblasts (Shiomi et al., 2011) or other immortalized human myogenic cell lines with similar properties
*Note: We know of four human immortalized myogenic cell lines (and derivatives thereof) described in the literature (Zhu et al., 2007; Shiomi et al., 2011; Thorley et al., 2016; Massenet et al., 2020). KD3 cells used here were initially obtained from N. Hashimoto. Inquiries for KD3 cells should now be addressed to Dr. Tohru Hosoyama, Dept. of Musculoskeletal Disease, The Geroscience Research Center, National Center for Geriatrics and Gerontology, 7-430 Morioka-cho, Obu City, Aichi Prefecture, Japan (toruhoso@ncgg.go.jp). Upon completion of an MTA, the cells might be obtained in Europe from the authors, or in the US from Dr. L. Weiss, Albert Einstein College of Medicine, 1300 Morris Park Avenue, Forchheimer Building, Bronx, NY 10461, USA (louis.weiss@einsteinmed.edu). LHCN-M2 cells (Zhu et al., 2007) are commercially available from Evercyte. Cells from Massenet et al. might be obtained from the respective authors.*

**Reagents**

1. Dulbecco’s modified Eagle’s medium (DMEM) (Gibco Life Technologies, catalog number: 12800-082)
2. Glucose (Carl-Roth, catalog number: X997.2)
3. L-glutamine (Thermo Fisher Scientific, catalog number: 25030081)
4. Sodium pyruvate (Capricorn Scientific, catalog number: NPY-B)
5. Penicillin/streptomycin (Capricorn Scientific, catalog number: PS-B)
6. Bovine serum, heat inactivated, iron fortified (Capricorn Scientific, catalog number: CS-IF-1A)
7. Fetal bovine serum, heat inactivated (Capricorn Scientific, catalog number: FBS-12A)
8. Fetal bovine serum, heat inactivated low endotoxin (Capricorn Scientific, catalog number: FBS-LE-12A)
9. Ultroser^®^ G (Cytogen, catalog number: 15950-017)
10. Donor horse serum (Capricorn Scientific, catalog number: DHS-1A)
11. Human recombinant insulin (PAN-Biotech, catalog number: P-2701001)
12. Insulin Lispro (Sanofi, catalog number: 12910612)
13. Human holo-transferrin (Sigma, catalog number: TO665)
14. Na_2_SeO_3_ (Sigma-Aldrich, catalog number: 214485)
15. RPMI 1640 pH 7.4 (containing 4 mM L-glutamine) (Sigma-Aldrich, catalog number: R1383)
16. HEPES (Sigma-Aldrich, catalog number: H3375)
17. DMSO (AppliChem, catalog number: A3672)
18. Collagen I from rat tail (Corning, catalog number: 354236)
19. NaCl (Merck catalog number: 1.06404)
20. KCl (Merck, catalog number: 1.04936)
21. KH_2_PO_4_ (Merck, catalog number: 1.04877)
22. Na_2_HPO_4_·2H_2_O (Merck, catalog number: 1.06580)
23. Acetic acid (Carl Roth, catalog number: 3738.4)
24. 0.05% trypsin/0.53 mM EDTA (Capricorn Scientific, catalog number: TRY-1B10)

**Media and solutions**

1. 70% ethanol for sterilization
2. HFF growth medium (see Recipes)
3. Tachyzoite medium (see Recipes)
4. Myoblast growth medium (see Recipes)
5. Myoblast differentiation medium (see Recipes)
6. Cyst medium (see Recipes)
7. Freezing medium (see Recipes)
8. Rat tail collagen type I (see Recipes)
9. 1× Trypsin-EDTA solution in PBS (w/o Ca^2+^ and Mg^2+^)
10. Phosphate-buffered saline (PBS) (w/o Ca^2+^and Mg^2+^)
*Note: All media have to be sterile.*

**Material**

1. T-25 or T-75 cell culture flasks with filter caps (TPP, catalog number: 90026 or 90076)
2. Microtiter plates 6 well or 96 well (TPP, catalog number: 92006 or 92048)
3. 15 mL and 50 mL conical centrifuge tubes (TPP, catalog number: 91015 and 91050)
4. 1.5 mL cryovials (Nalgene, catalog number: 5000-1020)
5. Pipette tips [10, 200, 1,000 µL (Various brands)]
6. Sterile syringe filters (0.22 μm) (TPP, catalog number: 99722)
7. C-Chip disposable hemocytometer (NanoEnTek Inc., DHC-N01) or Neubauer hemocytometer
8. Syringes (20 mL) (Luer Lock, Injekt, catalog number: 4606736V)
9. Blunt cannula (27 G) (Sterican, catalog number: 9180117)
*Note: Examples for providers/manufacturers are given; similar material from others will probably work as well.*

**Recipes**

1. **HFF growth medium**
  DMEM pH 7.2 (containing 3.75 g/L NaHCO_3_, 25 mM glucose, 4 mM L-glutamine, and 1 mM sodium pyruvate), supplemented with 1× penicillin/streptomycin and 10% bovine serum. Store at 4 °C.
2. **Tachyzoite medium**
  HFF growth medium but with only 1% fetal bovine serum. Store at 4 °C.
3. **Myoblast growth medium**
  DMEM pH 7.2 (containing 3.75 g/L NaHCO_3_, 25 mM glucose, 4 mM L-glutamine, and 1 mM sodium pyruvate), supplemented with 1× penicillin/streptomycin, 20% fetal bovine serum, and 2% Ultroser^®^ G. Store at 4 °C.
4. **Myoblast differentiation medium**
  DMEM pH 7.2 (containing 3.75 g/L NaHCO_3_, 25 mM glucose, 4 mM L-glutamine, and 1 mM sodium pyruvate), supplemented with 1× penicillin/streptomycin, 2% donor horse serum, 10 µg/mL human recombinant insulin (alternatively, Insulin Lispro), 5 µg/mL human holo-transferrin, and 10 nM Na_2_SeO_3_. Store at 4 °C.
*Note: Lower concentrations of insulin might also work, according to literature.*
5. **Cyst medium**
  RPMI 1640 pH 7.4 (containing 4 mM L-glutamine), supplemented with 5 mM glucose, 1× penicillin/streptomycin, 50 mM HEPES, 2% donor horse serum, 10 µg/mL human recombinant insulin (alternatively, Insulin Lispro), 5 µg/mL human holo-transferrin, and 10 nM Na_2_SeO_3_. Store at 4 °C.
6. **Freezing medium**
  90% heat-inactivated fetal bovine serum low endotoxin and 10% DMSO. Store at 4 °C.
7. **Rat tail collagen**
  Collagen I from rat tail dissolved at a stock concentration of 0.1 mg/mL in 100 mM acetic acid. Use 1:6.67 in water. Store at 4 °C.
8. **1× Trypsin-EDTA solution**
  0.05% trypsin/0.53 mM EDTA in PBS (w/o Ca^2+^ and Mg^2+^)
9. **Phosphate-buffered saline (PBS) without calcium and magnesium**
  MilliQ water supplemented with 137 mM NaCl, 2.7 mM KCl, 1.5 mM KH_2_PO_4_, and 6.5 mM Na_2_HPO_4_·2H_2_O at pH 7.4.

## Equipment

1. Humidified CO_2_ cell incubator at 36.7 °C, 10% CO_2 _(Thermo Fisher Scientific, model: Heracell 150i CO_2_ incubator)
2. Incubator at 36.7 °C, ambient CO_2_ (Binder Inkubator, model: KB 240)
3. Safety cabinet for BSL-2 work (Luft- und Reinraumtechnik GmbH, model: BDK S1200 laminar flow workbench)
4. Water bath at 37 °C (Memmert GmbH & Co. KG, model: WNB 14)
5. Adjustable micropipettes 2–20 µL, 20–200 µL, 100–1,000 µL (Eppendorf GmbH, model: Research Plus micropipette)
6. Pipetting aid for serological pipettes (Brand GmbH, model: Accu-Jet Pro pipette)
7. Vacuum aspirator system, Integra Biosciences Vacusafe Comfort Aspiration system (Integra, model: 10590273)
8. Inverted light microscope with phase contrast, differential interference contrast, or similar optics (Nikon, model: Eclipse Ts2 inverted microscope)
9. Centrifuge with swing-out rotor and refrigeration (Hettich GmbH & Co. KG, model: Rotina 380R centrifuge 1706)
10. CoolCell LX Cell Freezing Vial Containers (Fisher Scientific, Corning, catalog number: 15572771) or similar cryo-freezing container
11. Refrigerator 4 °C (AEG, model: RTB415E2AW)
-20 °C freezer (Liebherr, model: Mediline LGex 3410)
-70 °C freezer (SANYO, model: VIP Series MDF-U53V ultra-low temperature freezer)
14. Liquid nitrogen storage capabilities
*Note: Examples for providers/manufacturers are given; similar material from others will probably work as well.*

## Procedure

**General description of the procedure and notes**

**Figure 1.**
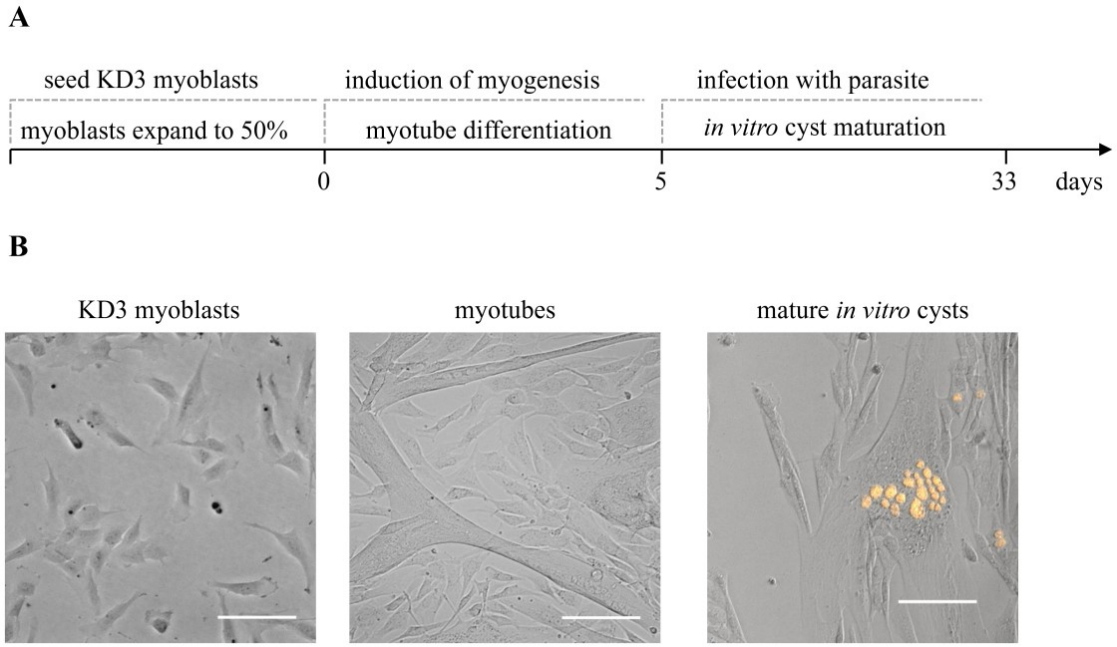
Overview of the protocol steps. (A) Timing of the cultivation of in vitro *T. gondii* cysts. KD3 myoblasts are seeded into desired cell culture vessels. When they reach a maximum confluency of 50%, differentiation is induced. After five days, the cells feature longer tube-like multinucleated cells, which are hallmarks of the myotube stage. Myotubes are infected with cystogenic *T. gondii* parasites. Over the course of four weeks of CO_2_ depletion, tachyzoites differentiate into bradyzoites and mature to in vitro cysts. (B) Representative pictures of the same culture at relevant developmental stages. Prugniaud parasites express the red fluorescent protein tdTomato. Scale bar indicates 100 µm.

Generating mature *T. gondii* cysts in KD3 human myotubes involves three steps (Figure 1): (i) set up a KD3 myoblast culture, (ii) differentiation of these cells into KD3 myotubes, and (iii) infection of myotubes with tachyzoites and the differentiation of parasites to bradyzoites. We also suggest experiments to confirm the maturation of cysts.

**(i) Myoblast culture**

**Figure 2.**
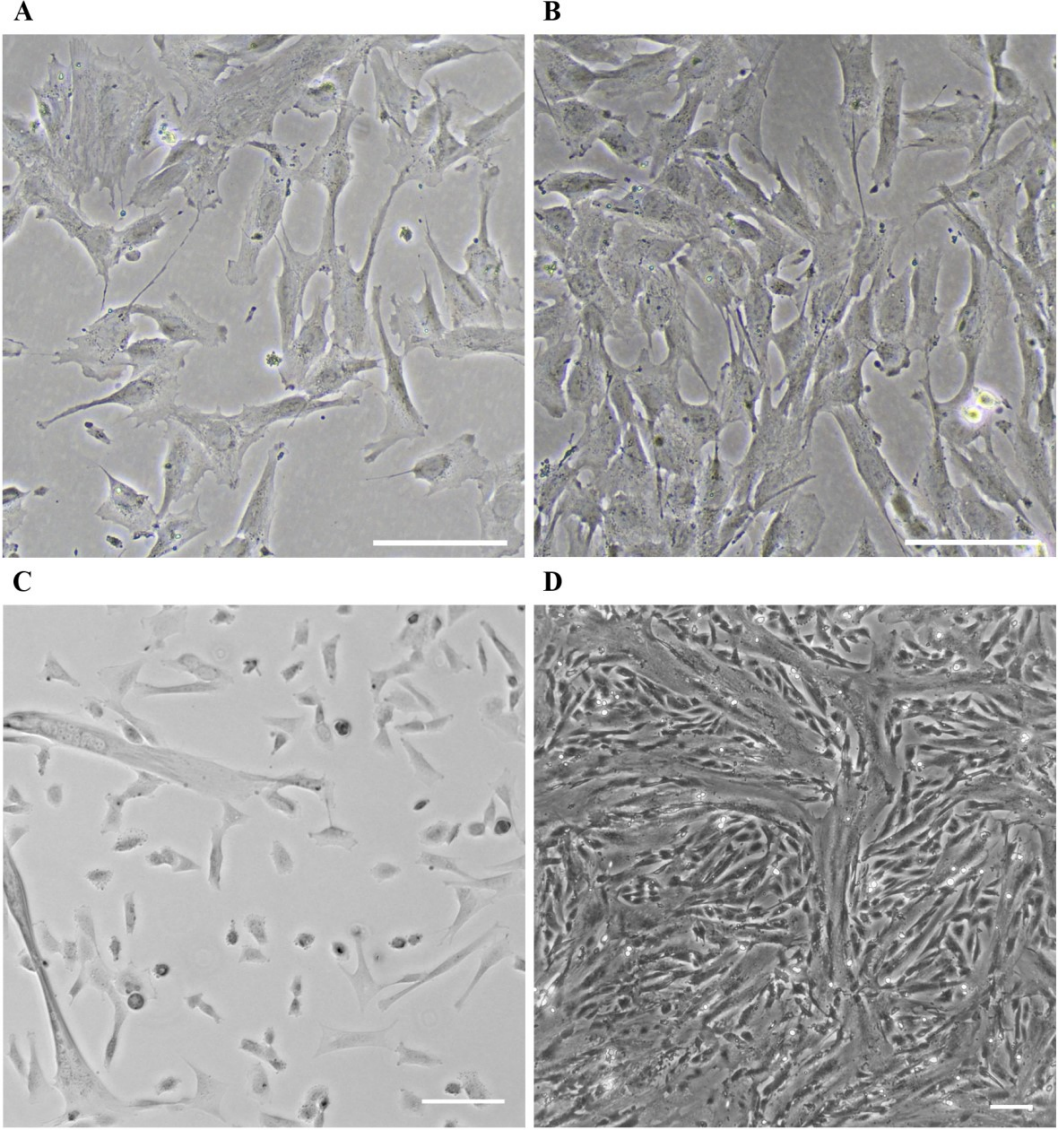
KD3 culture at various stages. (A) Healthy KD3 myoblast culture at the right confluency for myotube induction. Scale bar indicates 500 µm. (B) KD3 myoblast cultures at the maximum density allowed before splitting. (C) Improper handling or high passage number of the KD3 culture creates a mixture of myoblasts and emerging myotubes. Scale bar indicates 100 µm. (D) KD3 myotubes five days post induction. Scale bar indicates 100 µm.

Handling of KD3 myoblasts is adapted from the procedure described by Shiomi et al. (2011). KD3 myoblasts appear similar to endothelial cells (Figure 2A) and are slightly heat-sensitive (avoid prolonged temperatures higher than 37 °C during cultivation). During maintenance of the myoblasts, it is imperative to keep the main culture subconfluent (<70%), as cell-to-cell contact appears to act as a trigger for differentiation into myotubes. Before the monolayer reaches this density, the cells are subcultured (split). The medium is exchanged for warm phosphate-buffered saline without magnesium and calcium. Pre-warmed trypsin-EDTA solution is added to cover the monolayer and the culture is incubated at 36.7 °C until cells detach. The trypsinization reaction is stopped by adding myoblast growth medium, and a homogenous cell suspension is generated by pipetting. At this point, the cell density can be determined by counting. The suspension is distributed to suitable cell culture vessels, which can optionally be coated with collagen to enhance myotube attachment. Do not aim for a confluency below 5%, as this suppresses myoblast growth. Incubate cultures at 36.7 °C at 5%–10% CO_2_.

Since cell-to-cell contact cannot be prevented even at low confluency subculturing, KD3 myoblasts may unintentionally start to differentiate to myotubes, and the culture may exhibit a mixture of both cell types (Figure 2C). We often observe this with increased confluency; the growth rate drastically declines, and the culture becomes unsuitable for further use. The number of usable passages of the culture thus highly depends on handling, and we recommend monitoring cell behavior in correlation with the respective passage number. Generally, the number of passages should not exceed 16 of a freshly thawed vial. An occasional selection for the presence of the transgenes might be advisable. The cells are resistant to G418 (400 µg/mL) and puromycin (0.5 µg/mL) (Shiomi et al., 2011).

Long-term KD3 myoblast storage is done in liquid nitrogen in freezing medium. Use a controlled freezing rate apparatus or an isopropanol chamber to ensure a slow and steady temperature drop. For thawing, quickly thaw in a 37 °C water bath, replace the freezing medium with growth medium, and distribute cells to a cell culture vessel. Incubate at 36.7 °C at 5%–10% CO_2_ until the culture is ready for subcultivation. We usually use 0.3 mL of medium per cm^2^ cell culture vessel.

**(ii) Myoblast differentiation**

KD3 myoblasts are progenitor cells that undergo myogenesis when induced by serum starvation (Shiomi et al., 2011). Growth medium is replaced with myoblast differentiation medium at a confluency of 20%–50% (Figure 2A). Cells fuse and form multinucleated tubes over the course of five days. Often, myoblasts replicate once or twice before they fuse (Figure 2B). The differentiation efficiency depends on the general potency and passage number of the myoblast culture.

**(iii) Tachyzoite infection and bradyzoite maturation**

In general, every cystogenic strain of *T. gondii* can be used for infection, and a selection of type I, type II, and type III strains were tested directly for their ability to generate DBA-positive and SAG1-negative cysts (Christiansen et al., 2022). For a fully matured, in vivo–like cyst culture, we recommend the use of type II or type III strains. We frequently cultivate a Pru-∆hxgprt tdTomato (Chtanova et al., 2008) strain. Furthermore, we recommend using a fluorescent protein–expressing strain to easily monitor the cyst development, since the cysts may be difficult to find using brightfield or phase-contrast microscopy. It is also highly beneficial to use a tachyzoite strain that has been recently re-differentiated from either a cyst-containing tissue homogenate (Dubey, 1998) or a previous in vitro cyst culture, as long-term tachyzoite cultures lead to low differentiation efficiencies (Colos-Arango et al., 2023).

The myotube culture is infected with 7.2 × 10^3^ tachyzoites per cm^2^ surface area in cyst medium. The culture is incubated at 36.7 °C and ambient CO_2_ concentration. Ambient CO_2_ levels limit the rate of parasite pyrimidine biosynthesis and facilitate stage conversion (Bohne and Roos, 1997). After infection, the culture needs to be monitored daily and the medium is replaced every second to third day. Parasite stage-conversion depends on the quality of the myotubes and the cystogenicity of the strain and will start immediately post-infection. However, long-term tachyzoite cultures with a decreased cystogenic potential may require additional washing steps if parasite egress is apparent to prevent overgrowth of the culture with tachyzoites. After four weeks, the cysts are largely functionally mature (Christiansen et al., 2022).

Throughout the experiment, myotubes move and change their shape, which can lead to cell detachment. Furthermore, throughout the culture period, cysts grow in size, new cysts will emerge from egressed bradyzoites, and cysts may also dissipate.

**Bench protocol**

All parasite and muscle cell handling should be done in a sterile laminar flow BSL-2 workbench.

- **Thawing KD3 myoblasts**
  1. Warm myoblast growth medium to 37 °C in a water bath.
  2. Sterilize all involved surfaces of the laminar flow BSL-2 workbench with 70% ethanol.
  3. Place cryovial containing frozen KD3 myoblasts on ice.
  4. In the laminar flow BSL-2 workbench, fill a 15 mL centrifuge tube with warm myoblast growth medium.
  5. Place the cryovial in the 37 °C warm water bath until the outer layer of the frozen cell suspension starts to melt.
  6. Sterilize the cryovial with 70% ethanol.
  7. In the laminar flow BSL-2 workbench, transfer the cell suspension from the cryovial to the centrifugation tube.
  8. Centrifuge the suspension at 500× *g* for 5 min at room temperature.
  9. In the laminar flow BSL-2 workbench, open the centrifuge tube and discard the supernatant.
  10. Gently resuspend the pellet in 15 mL of myoblast growth medium. Transfer the suspension to a T-75 cell culture flask with a filter cap (choose flask size depending on cell density).
  11. Incubate in a humidified incubator at 5%–10% CO_2_ at 36.7 °C.
  12. The next day, visually inspect confluency and cell adhesion under a microscope.
- **Subculturing KD3 myoblasts**
  1. **Critical:** Before the monolayer reaches a confluency of 70%, subculture the myoblasts.
  2. Warm PBS (without Ca^2+^ and Mg^2+^) and 0.05% trypsin/EDTA in PBS to room temperature.
  3. Warm myoblast growth medium in a water bath to 37 °C.
  4. Sterilize all involved surfaces of the laminar flow BSL-2 workbench with 70% ethanol.
  5. Aspirate the culture medium.
  6. Wash the monolayer with at least 10 mL of pre-warmed PBS (without Ca^2+^ and Mg^2+^).
  7. Add 0.02 mL/cm^2^ trypsin/EDTA in PBS to the monolayer.
  8. Incubate at 36.7 °C and visually inspect for the detachment of cells (approximately 2 min).
  9. Add sufficient myoblast growth medium to neutralize trypsin activity and mix the suspension by pipetting. Transfer the suspension into a centrifuge tube and centrifuge at 500× *g* for 5 min at room temperature.
  10. Discard the trypsin-containing supernatant and add myoblast growth medium (0.3 mL per cm^2^ culture area).
  11. **Optional:** Use a hemocytometer to determine the cell density and distribute 6 × 10^3^ cells per cm^2^ culture area into desired cell culture vessels after the trypsinized cells are spun down and resuspended in fresh growth medium.
  12. Incubate in a humidified incubator at 5%–10% CO_2_ at 36.7 °C. Typically, 70% confluency is reached after two to three days.
- **Freezing KD3 myoblasts**
  1. **Critical:** Use a culture with a low passage number for cryopreservation.
  2. **Critical:** Freeze myoblasts before the monolayer reaches a confluency of 70%.
  3. Equilibrate PBS (without Ca^2+^ and Mg^2+^) and trypsin/EDTA in PBS to room temperature.
  4. Warm freezing medium in a water bath to 37 °C.
  5. Sterilize all surfaces involved with 70% ethanol.
  6. Discard the medium.
  7. Wash the monolayer with at least 10 mL of PBS (without Ca^2+^ and Mg^2+^).
  8. Add 0.02 mL/cm^2^ trypsin/EDTA in PBS to the monolayer.
  9. Incubate at 37 °C and visually inspect for the detachment of cells (approximately 2 min).
  10. Add freezing medium to stop trypsin digestion and gently mix the suspension by pipetting.
  11. Transfer the suspension into a centrifuge tube and centrifuge the cell suspension at 500× *g* for 5 min at room temperature. Discard the trypsin-containing supernatant and add myoblast growth medium.
  12. Aliquot 75 cm^2^ worth of cells per cryovial (approximately 1.5–1.8 × 10^6^ cells).
  13. Place cryovials in a cryo-freezing container and freeze them overnight at -70 °C.
  14. Store cryovials in liquid nitrogen.
- **Differentiating KD3 myoblasts into myotubes**
  1. **Optional:** Coat the desired cell culture vessel with 15 μg/mL rat tail collagen type I for 1 h at room temperature. Wash with PBS three times or leave until completely dry in the laminar flow BSL-2 workbench. Coated plasticware may be stored at 4 °C for a few weeks.
  2. Follow the instructions of Section B, steps 1–10.
  3. Plate 6 × 10^3^ cells per cm^2^ culture area into the desired culture vessel containing myoblast growth medium.
  4. Incubate in a humidified incubator at 5%–10% CO_2_ at 36.7 °C.
  5. **Critical:** Visually inspect confluency. Begin differentiation at 20%–50% monolayer confluency (usually after one or two days).
  6. Warm myoblast differentiation medium to 37 °C in a water bath.
  7. Sterilize all surfaces involved with 70% ethanol.
  8. Aspirate growth medium. Make sure to completely remove the myoblast growth medium by washing the cells with warm PBS at least once.
  9. Add myoblast differentiation medium.
  10. Incubate in a humidified incubator at 5%–10% CO_2_ at 36.7 °C.
  11. After five days, the cells have fused and formed multinucleated myotubes.
  12. **Optional:** Confirm cell fusion by appropriate staining protocols (e.g., immunofluorescence staining for myosin heavy chain and nuclei) and calculate myogenic index (Noë et al., 2022).
- **Infection of KD3 myotubes and cyst maturation**
  1. Tachyzoites are maintained in HFF host cells using standard procedures (Khan and Grigg, 2017).
  2. Determine the parasite count in a freshly egressed or syringe-released tachyzoite suspension.
  3. The myotubes are infected with 7.2 × 10^3^ tachyzoites per cm^2^. Prepare an appropriate dilution of the parasite suspension in cyst medium.
  4. Wash the myotube monolayer with cyst medium once and then apply the parasite suspension.
  5. **Critical:** Incubate in an incubator at 36.7 °C at ambient CO_2_ concentration.
  6. Change cyst medium gently every second to third day. Leave 25% of the volume in the cell culture vessel and slowly replace only 75% to avoid perturbation of the monolayer.
  7. **Critical:** Visually monitor the culture using an inverted microscope. Inspect for possible tachyzoite overgrowth, myotube delamination, or cyst loss (see troubleshooting section).
  8. After four weeks, the culture contains matured cysts.
  9. **Optional:** To induce tachyzoite re-differentiation, replace cyst medium with tachyzoite medium and incubate in a humidified incubator at 5%–10% CO_2_ at 36.7 °C.

## Validation of protocol

Several markers are known to indicate bradyzoite development, including the presence of proteins BAG1, LDH2, and p21, and the absence of the SAG1 surface protein. The formation of the cyst wall may be monitored using antibodies against CST1 and CC2 or *Dolichos biflorus* agglutinin (DBA) staining (Yang and Parmley, 1995; Gross et al., 1996; Dubey et al., 1998; Knoll and Boothroyd, 1998; Zhang et al., 2001). All these markers start to appear within the first week of cyst maturation and have been shown to develop even using fibroblast-based methods. In contrast, functional cyst maturation requires more time. Resistance to pepsin, temperature stress, and drugs, as well as oral infectivity gradually emerge after 14 days and continue to develop until at least 28 days (Christiansen et al., 2022). Drug resistance can be easily monitored by exposing cysts for one week to high doses of pyrimethamine (20 µM) and sulfadiazine (20 µM) and monitoring re-differentiation.

## General notes and troubleshooting

**Application of the protocol**

The method can be helpful in many applications, considering the different characteristics to be studied and the scale required for their analysis. While the cyst wall starts to develop within the first week of differentiation as seen by DBA staining (Christiansen et al., 2022), antifolate tolerance fully developed only after four weeks. Further, resistance to pepsin digestion is increasing throughout four weeks of maturation. Myotubes are a natural host cell type for *T. gondii* and thus may provide a more relevant system to investigate parasite interactions with the host cell than, for instance, HFF.

Downstream applications require suitable cyst isolation protocols. We performed an LC-MS-driven metabolic characterization of in vitro bradyzoites. To this end, we isolated tissue cysts using DBA- and streptavidin-coated magnetic beads from syringe-passaged infected KD3 myotubes (Christiansen et al., 2022). This isolation procedure can be performed at 4 °C and does not require detergents, polymers, or other MS-incompatible chemicals.

For other applications such as imaging, intact cysts can also be isolated using a percoll gradient (Watts et al., 2017). Single bradyzoites have been obtained using either pepsin (Dubey, 1998) or trypsin digestion (Jacobs et al., 1960; Fu et al., 2021). Enzymatic treatment may be directly applied to the monolayer and may provide a means of removing immature bradyzoites before purification.

Given the need for new drugs and drug targets, research efforts in the field of drug discovery have intensified over the last years (Dittmar et al., 2016; Adeyemi et al., 2018; Spalenka et al., 2018; Han et al., 2020). However, none of these studies integrated matured cysts into the screening process. Our method can be adapted into a 96-well plate format suitable for drug screens. Compounds that are cidal to bradyzoites would prevent regrowth of re-differentiated tachyzoites, which are easily detectable by fluorescence or plaque assay. In vitro bradyzoites develop tolerance towards antifolates and other antiparasitics in a time-dependent manner (Christiansen et al., 2022).

In the last 10 years, more than 135 studies in PubMed have mentioned the use of mice in the context of *T. gondii* bradyzoites. Assuming an average of 100 mice per study and an unknown number of mice that are routinely infected to have a constant supply of tissue cysts, this adds up to several thousand animals. A significant number of those could have been replaced if adequate in vitro systems like this one would have been applied. We hope that this protocol encourages the community to consider its usefulness instead of mouse experiments, where appropriate, thereby contributing to the 3R principle.

**Limitations and further development of the procedure**

**Improving the myotube culture**

Terminally differentiated myotubes are a suitable cell type for obtaining mature tissue cysts since they are long-lived under in vitro conditions. However, occasionally we observe the loss and morphological changes of myotubes, which may have a negative impact on cyst yield. Infected and detached myotubes might be rinsed off with media change. This problem may be addressed by improving host cell attachment to the culture vessel using various methods (Madden et al., 2015; Bettadapur et al., 2016; Denes et al., 2019; Martins et al., 2022). Finally, cellular senescence may also pose a limit on the maximal passages of KD3 cells and may be ameliorated by reversine treatment (Rajabian et al., 2023). Mild heat stress from temperature fluctuations of cell culture incubators may also induce differentiation and needs to be avoided (Yamaguchi et al., 2010). Whether any of these strategies provide improvements for in vitro cyst generation needs to be tested.

**Improving the cyst culture**

A healthy, long-lasting myotube culture is required but not sufficient for in vitro cyst maturation. In particular, parasite strains that have been passaged over extended periods of time as tachyzoites can easily overgrow in the first week of infection (Colos-Arango et al., 2023). Adjusting the multiplicity of infection can ameliorate this problem. Ideally, parasites intended for in vitro bradyzoite generation that have been recently isolated from an infected mouse brain or a previous long-term bradyzoite culture should be used.

Over the course of a four-week culture, the medium is changed 9–12 times, and associated shearing forces may lead to the loss of some cysts. This can be prevented by careful handling and minimizing medium changes in general. We have not experimented with chemostat culture or automated pipetting systems (Pazdzior and Kubicek, 2021). They may provide a more stable and predictable environment during the whole culturing period and could reduce workforce for large-scale approaches.

Cyst maturity can be assessed by a variety of assays relevant to the application. To date, there is a lack of very late reporter strains that allow monitoring the expression of late (>2 weeks) bradyzoite markers. However, bradyzoites within tissue cysts are considered to form a heterogeneous population and may thus display different proteins (Watts et al., 2015). The reported proteomic datasets from up to 5-month-old bradyzoites from infected mouse brains could provide a starting point for the construction of such reporter strains (Garfoot et al., 2019).

**Reduction of animal ingredients and cost-effectiveness**

To satisfy 3R principles and reduce costs, it is preferable to reduce both the use of animal experiments and the use of animal-derived products in cell culture. In particular, serum components in media formulations used in this protocol may be replaced or reduced. Serum-free medium maintains murine C2C12 cells in both their myoblasts and myotube stage, respectively (Jang et al., 2022). In particular, myotube formation can also be induced in serum-free, BSA-supplemented media for human skeletal muscle cell isolates (Rajabian et al., 2021). The impact of these serum-reduction strategies on cyst maturation and their resemblance to in vivo cysts, however, remains to be established.

**Troubleshooting**

**KD3 myoblasts do not completely differentiate into myotubes**

KD3 myoblasts may be used up to 16 passages upon thawing a new vial. However, we observed that with increased passage numbers their potential to differentiate into myotubes decreases and myotube formation may take longer. Also, myotube formation is effectively induced by serum starvation, and high-serum myoblast growth medium has to be removed completely. Myoblasts exhibit the potential to differentiate into other cell types besides myotubes such as adipocytes or osteocytes depending on the media composition (Shiomi et al., 2011). If the differentiation of myotubes was only partially successful, and, for example, a subsequent treatment of the cyst culture involves fatty acids, uncommitted myoblasts will differentiate to adipocytes.

**KD3 myotube monolayer has gaps**

Delamination of myotube monolayers can occur; however, not to an obstructive extent. Clusters of detaching myotubes may leave empty patches and incompletely differentiated myotubes behind. We found this phenomenon to be more prevalent when myoblasts were grown too dense prior to myogenesis induction. Seed fewer myoblasts or induce myogenesis earlier.

**Parasites do not differentiate properly (tachyzoite overgrowth)**

After infection, the parasitophorous vacuole forms normally, but tachyzoite growth is not quenched. Parasites continue their lytic cycle, thus overgrowing the whole culture. We encountered such behavior in particular with lab-adapted strains. To an extent, washing the monolayer more thoroughly in the first week post-infection and lowering the tachyzoite inoculum helps to avoid excess tachyzoites. If parasites recently isolated from mouse brain tissue are not available, gradual in vitro adaptation to bradyzoites enhances their ability to form cysts. These bradyzoites can either be used for infection directly or be re-differentiated into tachyzoites with higher cyst-forming capacity. It is advisable to generate freezer stocks of suitable tachyzoite cultures to ensure reliable and stable differentiation behavior. If parasite overgrowth cannot be avoided by the aforementioned measures, pharmaceutical inhibition of tachyzoite growth might help to allow bradyzoites to mature for longer. In that case, we recommend the use of 10 nM buparvaquone, a *bc*_1_ complex inhibitor.

**Cyst numbers vary**

Many variable host- and parasite-related factors influence this in vitro system. The comparatively long culture periods amplify these effects and lead to significant differences in total cyst numbers. Try to work as consistently as possible. Ensure that the myoblasts passage number is not too high, the myoblast confluency does not exceed 70% before differentiating them to myotubes, the monolayer has no gaps, the parasites are freshly egressed or released via syringing before infection, and that the parasites have not been cultured as tachyzoites for extended times. The latter is especially important when comparing bradyzoite formation efficiencies of different parasite strains. Using this protocol and a cystogenic parasite strain, approximately 10^6^ cysts can be obtained from a T150 dish (Christiansen et al., 2022). After maturation, we occasionally observe multiple cysts per cell but few infected cells.

**Myotubes detach from glass coverslips**

KD3 myotubes can be grown and infected on glass coverslips for microscopy applications. We observe an increased rate of detachment of myotubes in these cultures. This may be compensated by coating the coverslips with rat collagen (see above), low levels of Cell-Tak, or gelatine.
